# Niche signals and transcription factors involved in tissue-resident macrophage development

**DOI:** 10.1016/j.cellimm.2018.02.005

**Published:** 2018-08

**Authors:** Wouter T'Jonck, Martin Guilliams, Johnny Bonnardel

**Affiliations:** Laboratory of Myeloid Cell Ontogeny and Functional Specialization, VIB-UGent Center for Inflammation Research, Technologiepark 927, 9052 Gent, Belgium; Department of Biomedical Molecular Biology, Ghent University, Technologiepark 927, 9052 Gent, Belgium

**Keywords:** CNS, central nervous system, DC, dendritic cell, KO, knockout, LC, Langerhans cell, NE, norepinephrine, OPG, osteoprotegerin, TF, transcription factor, Developmental immunology, Macrophage development, Niche, Tissue-resident macrophage, Transcription factors

## Abstract

Tissue-resident macrophages form an essential part of the first line of defense in all tissues of the body. Next to their immunological role, they play an important role in maintaining tissue homeostasis. Recently, it was shown that they are primarily of embryonic origin. During embryogenesis, precursors originating in the yolk sac and fetal liver colonize the embryonal tissues where they develop into mature tissue-resident macrophages. Their development is governed by two distinct sets of transcription factors. First, in the pre-macrophage stage, a core macrophage program is established by lineage-determining transcription factors. Under the influence of tissue-specific signals, this core program is refined by signal-dependent transcription factors. This nurturing by the niche allows the macrophages to perform tissue-specific functions. In the last 15 years, some of these niche signals and transcription factors have been identified. However, detailed insight in the exact mechanism of development is still lacking.

## Highlights

• Pre-macrophages share a core program established by lineage-determining transcription factors.
• Signal-dependent transcription factors adapt the core program.
• Niche signals instruct pre-macrophages to perform tissue-specific functions.
• One organ may contain multiple niches and different organs may contain similar niches.

## 1 Introduction

Tissue-resident macrophages were first described by the Russian scientist Élie Metchnikoff in 1883 [1]. These cells are present in all tissues of the body where they form a first line of defense against pathogens and play an essential role in maintaining tissue homeostasis [2], [3]. In the past decade, new insights have been gained in the origin of tissue-resident macrophages. Briefly, they are derived from three progenitors, being yolk sac macrophages, fetal liver monocytes and circulating monocytes, which colonize the tissues in consecutive waves (Reviewed in [4], [5]).

Tissue-resident macrophages share several common features such as the ability to phagocytize particles, pathogens and dying cells, initiate immune responses through the production of cytokines and chemokines and the expression of markers such as CD11b, F4/80 and CD64 which are often found on the cell surface of murine tissue-resident macrophages [6], [7], [8], [9], [10]. These features are part of a core macrophage program which is largely shared by all tissue-resident macrophages. Next to these common features, each macrophage population has a unique identity and function. Interestingly, this functional specialization is dependent on the tissue in which they reside. For example, it has been shown recently that cardiac macrophages facilitate electrical conduction through Cx43-containing gap junctions with cardiomyocytes [11]. By contrast, tissue-resident macrophages located in the brain, called microglia, are small star-shaped cells with an extensive lamellipodial network and while they are involved in brain surveillance by constantly probing the cellular environment, they are also crucial for brain development and homeostasis by regulating the synaptic pruning during postnatal development [12], [13], [14]. Another example are the lung alveolar macrophages which are involved in the clearance of alveolar surfactant [15]. The tissue-specific function of these macrophages implies that they must have a different functional identity. This functional specialization is governed by tissue-specific signals which regulate the expression or activity of signal-dependent transcription factors (TFs). In turn, these TFs adapt the core macrophage program by activating functional modules, which gives macrophages their functional identity.

In this review, we will first briefly touch upon the major lineage-determining TFs that establish the core macrophage program. Second, we will discuss the signal-dependent TFs which adapt this core program in response to environmental cues, allowing macrophage to perform tissue-specific functions.

## 2 Lineage-determining transcription factors and the core macrophage program

Macrophages form a very diverse group of mononuclear phagocytes. Despite this heterogeneity, a large transcriptional network and epigenetic landscape is shared among all macrophage populations [16], [17], [18]. This core macrophage program is established by a group of lineage-determining TFs which perform a general role in myelo-monocytic development by determining stem cell fate.

One of the most well studied master regulators in macrophage development is PU.1, which is regulated by RUNX1 (also known as AML1) [19], [20]. During the early stages of myeloid cell development, PU.1 determines myeloid progenitor fate in a concentration-dependent manner. A high amount of PU.1 leads to the development of macrophages whereas a low level of PU.1 is necessary for B cell development [21]. This concentration-dependent effect can be attributed to the numerous low- and high-affinity PU.1 binding sites present in the genome [22]. The low-affinity binding sites are only bound by PU.1 when a certain threshold concentration is exceeded. The developmental role of PU.1 is not restricted to macrophages and B cells. For example, PU.1 also regulates dendritic cell (DC) development in a concentration dependent manner through regulation of *Flt3* expression [23]. One of the major target genes of PU.1 in macrophage development is *Csf1r* [20], which encodes the receptor for interleukin-34 (IL-34) and monocyte colony-stimulating factor (M-CSF). IL-34 is specifically required for the development and maintenance of microglia and Langerhans cells [24], [25], whereas M-CSF is involved in survival, proliferation and maintenance of most other mononuclear phagocytes [26]. Together, PU.1 and *Csf1r* are essential for the formation of yolk sac macrophages [27]. Generally, PU.1 is involved in tissue-resident macrophage development by acting as a scaffold for histone modifiers which establish an enhancer landscape [28]. In addition, many TFs involved in tissue-resident macrophage development, function and activation perform their function through interaction with PU.1. For instance, it was shown that c-Jun can enhance the ability of PU.1 to drive expression of M-CSFR [29]. In addition, *Zeb2* has been recently described as being involved in M-CSFR regulation in the bone marrow [30] and has been proposed to be part of the core macrophage program since it is expressed in pre-macrophages, but this still remains to be validated [16].

Upon terminal differentiation, MafB is expressed by tissue-resident macrophages causing them to exit the cell cycle [31]. MafB, together with c-Maf, desensitize macrophages from the proliferative effect of M-CSF by inhibiting the expression of self-renewal genes such as *Myc*, *Klf2* and *Klf4* [32]. This happens through direct inhibition of macrophage enhancers, including PU.1. In self-maintaining tissue-resident macrophage populations, the inhibition of these enhancers can be temporarily lifted, allowing differentiated tissue-resident macrophages to re-enter the cell cycle [32]. Contrary to regenerative processes, this is not accompanied by dedifferentiation of the tissue-resident macrophages [31], [33]. In addition, MafB is essential for F4/80 maturation [34] and is involved in actin remodeling [35].

Other lineage-determining TFs have been proposed, including *Batf3*, *Pparg*, *Irf8* [16]. It is however not clear whether these factors are strictly needed for macrophage development. Moreover, it is unknown whether macrophages require continuous expression of these factors for their maintenance, survival or function.

Together, these lineage-determining TFs establish the core macrophage program during the pre-macrophage stage. This core program includes *Cx3cr1*, pattern recognition receptors, phagocytic receptors, Fcγ receptors (e.g. *Fcgr1*, encoding CD64), *Sirpα*, *Iba1*, *Mertk* and *Adgre1* (F4/80) which are expressed by almost all macrophage populations [7], [16], [36], [37]. Additionally, these lineage-determining TFs shape the epigenome and form an anchor point for signal-dependent TFs.

## 3 Niche signals and signal-dependent transcription factors

**Fig. 1:**
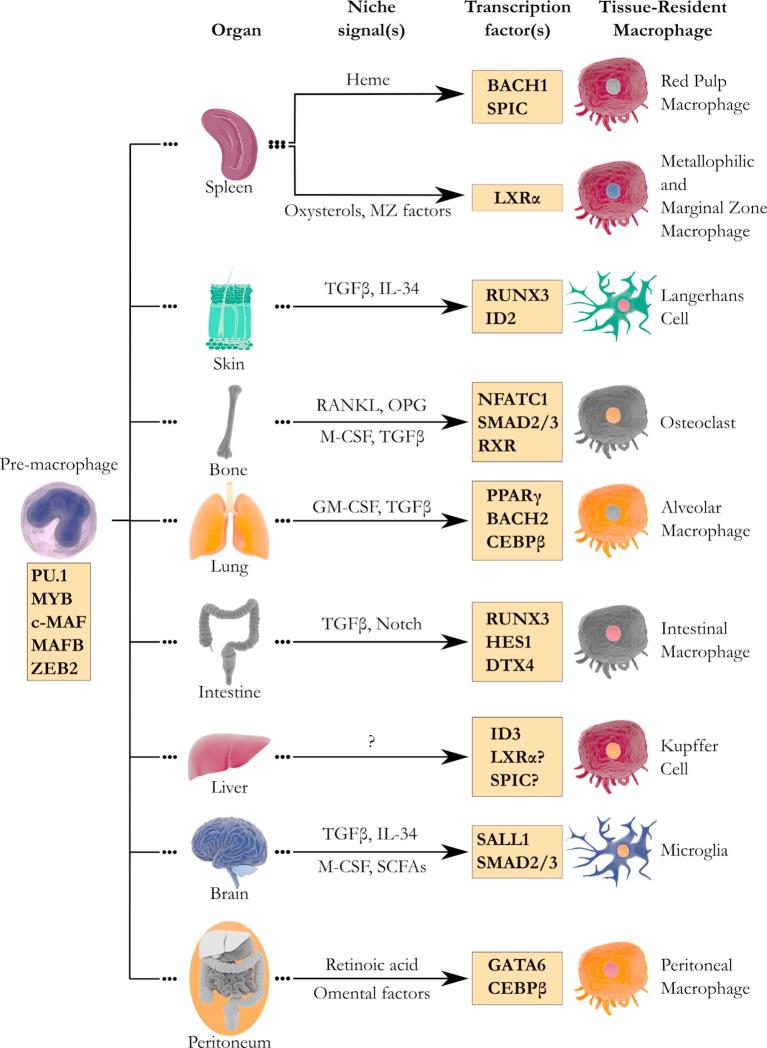
Lineage-determining and signal-dependent transcription factors involved in macrophage development; Macrophage development is regulated by two sets of transcription factors. First, a set of lineage-determining transcription factors, including, PU.1, MYB, c-MAF, MAFB and ZEB2, establish a core macrophage program which is largely shared by all macrophages. In response to niche signals, signal-dependent transcription factors adapt this core program, which gives tissue-resident macrophages their unique functional identity. MZ, marginal zone; TGFβ, transforming growth factor β; IL-34, interleukin 34; RANKL, Receptor activator of nuclear factor kappa-B ligand; OPG, osteoprotegerin; GM-CSF, granulocyte-macrophage colony-stimulating factor; M-CSF, monocyte colony-stimulating factor; SCFAs, short chain fatty acids [Illustrations of organs and cells were provided by Summersault 1824 (CC BY-NC-SA 4.0)].

Despite many similarities, macrophage identity and function are very diverse and unique for each tissue [6]. This implies that the core macrophage program, established during early development, has to be adapted in a tissue-dependent manner. According to the niche hypothesis [38], each macrophage is located in a particular niche which offers physical support and nurtures the cell through production of niche signals. These niche signals may include cytokines, metabolites and cell-cell contacts which initiate tissue-specific transcriptional networks in the pre-macrophages upon engraftment by driving signal-dependent TF expression or activation [39]. These signal-dependent TFs work in concert with lineage-determining TFs to refine the core macrophage program and imprint a transcriptional program in the tissue-resident macrophage to meet tissue-specific needs. This is done through direct activation of signature genes or by inducing chromatin remodeling which enables signal-dependent TFs to active signature genes [17], [18], [37], [39]. These signature genes are often required for the functional maturation and/or survival of tissue-resident macrophages. In this section, we will give an overview of the niche signals and their corresponding signal-dependent TFs in different macrophage populations (Fig. 1).

### 3.1 Red pulp macrophages

The spleen contains multiple subsets of macrophages, among them red pulp macrophages located in the red pulp of the spleen. They play a vital role in the clearance of senescent red blood cells, induction of regulatory T cell differentiation and protection against parasites through production of type I interferon [40], [41], [42], [43], [44]. Many advances have been made on signal-dependent TFs regulating the differentiation of these macrophages, among them the discovery of the essential role of SPIC in their development.

SPIC is a PU.1-related transcription factor which is highly expressed by red pulp macrophages, bone marrow macrophages and part of the F4/80^hi^ liver macrophages [41], [45]. Kohyama et al. have shown that *Spic*^−/−^ mice have a cell-autonomous defect in the development of red pulp macrophages that can be reverted by retroviral SPIC expression in bone marrow cells [41]. Of note, no defects were observed in monocytes or other macrophage populations. Heme, a metabolite of erythrocyte degradation, was shown to be sufficient to induce *Spic* in bone marrow-derived macrophages. At steady state, red pulp macrophages continuously phagocytize senescent or damaged erythrocytes to recycle the iron from the heme-containing hemoglobin. Consequently, pathological depletion of red pulp macrophages leads to an accumulation of heme in the spleen. While *Spic* expression in monocytes is constitutively inhibited by BACH1, the presence of heme induces its proteasomal degradation, thereby allowing SPIC to be expressed by monocytes that will reconstitute the red pulp macrophage population [45]. Other genes repressed by BACH1 include ferroportin-1 (*Fpn1*), which is involved in iron export [46] and heme oxygenase-1 (*Hmox1*), essential for heme catabolism [47]. In addition to being essential for red pulp macrophage function, HMOX1 is critical for their survival, as accumulation of heme is cytotoxic [48]. Thus, in the *Spic*^−/−^ deficient mouse model the inability of macrophages to express SPIC in the red pulp may hinder their capacity to perform splenic red pulp-specific functions, rendering them unable to survive in the red pulp. In essence, SPIC is important for both the functional maturation of red pulp macrophages and their survival. The discovery of heme as the driver of red pulp macrophage development was the first time a metabolic-driven differentiation of macrophages was described [45].

### 3.2 Marginal zone macrophages and metallophilic macrophages

Next to red pulp macrophages, the spleen also contains marginal zone macrophages and metallophilic macrophages [49]. Both are located in the marginal zone of the spleen, where they play a major role in the early control of blood-borne antigens [50] and can be distinguished from each other based on the expression of different surface receptors: SIGN-R1 [51] and MARCO [52] for marginal zone macrophages; CD169 for metallophilic macrophages [53]. Liver X receptor α (LXRα), encoded by the gene *Nr1h3*, is a transcription factor which plays a key role in the control of sterol homeostasis [54]. Gonzalez et al. have shown that this TF is essential for marginal zone macrophage and metallophilic macrophage differentiation as LXRα-deficient mice lack both macrophage populations [55]. Similar to other signal-dependent TFs, this effect is cell-intrinsic suggesting that the niche signal, which is potentially oxysterols, is still present in these LXRα-deficient mice [56]. Interestingly, LXRα deficiency does not affect the abundance of F4/80^+^ cells in the spleen during embryonic development, suggesting that LXRα is not essential to establish the core macrophage program but is crucial during the second, signal-dependent, phase of macrophage development. However, additional marginal zone signals might be necessary to facilitate the functional maturation of marginal zone macrophages, as hyper-activation of LXRα does not induce the marginal zone macrophage program in other splenic macrophages [55].

### 3.3 Large peritoneal macrophages

The peritoneum contains two types of macrophages, small peritoneal macrophages and large peritoneal macrophages, which can be distinguished from each other based on their differential expression of CD11b, F4/80 and MHCII [57]. In addition, whereas small peritoneal macrophages are short-lived and monocyte-derived, large peritoneal macrophages are thought to be primarily of embryonic origin, requiring little hematopoietic input in steady-state [58]. However, it was shown that short-lived peritoneal macrophages act as precursors of long-lived peritoneal macrophages which displace the embryonically derived population with age [59]. Recently, the role of GATA6, a large peritoneal macrophage-specific zinc finger-containing transcription factor, in large peritoneal macrophage maturation and function was described [60]. Okabe and Medzhitov showed that GATA6 is necessary for the activation of peritoneal macrophage-specific genes, including TGFβ2 which promotes IgA class switching in peritoneal B-1 cells. Transcriptional activation of *Gata6* is regulated by a two-step process. First, silencing H3K27me3 histone modifications are removed from the *Gata6* promotor. Second, retinoic acid present in the omentum binds the retinoic acid receptor which in turn binds the poised *Gata6* promotor [60]. *Ex vivo* cultured large peritoneal macrophages lose their expression of GATA6 [61]. This can be restored by adding retinoic acids to the culture medium, which suggests that this vitamin A metabolite is an important niche factor that regulates *Gata6* [60]. In addition, mice fed with a vitamin A-deficient diet showed a reduction in GATA6 expression and a reduced abundance of large peritoneal macrophages [60]. Together, these experiments showed the essential role of the retinoic acid in large peritoneal macrophage development and the influence of diet on the immune system. In essence, its presence in the omentum niche is necessary to drive expression of GATA6 which in turn activates peritoneal macrophage-specific genes. However, other omental factors might be necessary to refine the core macrophage program in a peritoneal macrophage-specific way, as *ex vivo* stimulation with retinoic acid can restore the expression of GATA6 and its effector genes, including some but not all peritoneal macrophage-specific genes [60]. Thus, not all peritoneal macrophage-specific genes are GATA6 dependent, implying that a combination of signals and transcription factors are involved in establishing the peritoneal macrophage program. TFs driving GATA6-independent genes may include RARβ and NFE2 [60]. Although GATA6 is not crucial for development, it plays a role in large peritoneal macrophage localization, proliferation, survival and functional maturation [60], [61], [62].

Another transcription factor involved in large peritoneal macrophage development is CEBPβ. CEBPβ KO mice exhibit elevated numbers of small peritoneal macrophage-like cells, but lack functional large peritoneal macrophages and alveolar macrophages [58]. Adoptive transfer experiments showed that wild type small peritoneal macrophages can differentiate into large peritoneal macrophages in these CEBPβ KO mice but not in wild type hosts [58]. The authors suggest that there may be a difference in the peritoneal microenvironment, potentially due to the presence or absence of endogenous large peritoneal macrophages. Indeed, these findings fit with the niche hypothesis [38]. Since the peritoneal niche is not full in CEBPβ KO mice, wild type small peritoneal macrophage can differentiate into large peritoneal macrophages which fill the peritoneal niche.

### 3.4 Kupffer cells

Kupffer cells form one of the largest tissue-resident macrophage population in the body [63]. They are located in the hepatic sinusoids and are involved in many homeostatic and immune functions such as iron, bilirubin and cholesterol metabolism, clearing gut-derived toxins and pathogens from the blood as well as removal of damaged erythrocytes and hepatocytes through expression of a large repertoire of scavenger receptors [64], [65]. Recently, the Geissmann group showed that inactivation of *Id3* impairs the development of liver macrophages [16]. *Id3*^−/−^ mice have reduced numbers of macrophages in the liver and head during embryonic development, which persists in the liver after birth [16]. Interestingly, *Id1* is upregulated in Kupffer cells lacking *Id3*, suggesting that ID1 might partially take over the role of ID3 [16]. ID1 and ID3 are both TGFβ-regulated TFs [66] and their expression pattern largely overlaps during embryogenesis [67], which could indicate that TGFβ might be one of the niche factors that drives Kupffer cell development. It is important to note that this study only relied on F4/80 to identify Kupffer cells. However, it was recently described that the F4/80^hi^ macrophage population in the liver can be subdivided in three maturation states based on expression of CLEC4F and TIM4 with bona fide Kupffer cells being CLEC4F^+^TIM4^+^ [64]. Other TFs linked to tissue-resident macrophage development include LXRα and SPIC, although these might be necessary for functional specialization, as no apparent defect in Kupffer cell abundance has been described in KO models involving these TFs [45], [55]. This was however also concluded based on F4/80 expression, so it may be required to revisit these findings using more specific Kupffer cell markers such as CLEC4F and TIM4.

### 3.5 Alveolar macrophages

Alveolar macrophages are located in the pulmonary alveoli and play a major role in the maintenance of alveolar homeostasis by clearing lipoprotein-containing alveolar surfactant produced by alveolar type II epithelial cells. They are derived from fetal liver monocytes that differentiate into alveolar macrophages during the first weeks of life following exposure to granulocyte-macrophage colony-stimulating factor (GM-CSF) produced by alveolar type II epithelial cells [16], [68]. The essential role of GM-CSF in pulmonary homeostasis was first shown in GM-CSFR KO mice which suffer from pulmonary alveolar proteinosis, a disease characterized by an accumulation of alveolar surfactant [69]. Recently, it was shown that this is due to a defect in alveolar macrophage development [68], [70]. As mentioned in the introduction, tissue-resident macrophages derive from three different progenitors. However, independent adoptive transfer of those progenitors into GM-CSFR KO mice restored the alveolar macrophage population, thereby showing the essential role of GM-CSF and the dominant role of the niche on tissue-resident macrophage development [71]. Binding of GM-CSF to the GM-CSFR on fetal monocytes leads to the expression of PPARγ [72]. In turn, PPARγ drives expression of genes involved in lipid metabolism, storage and degradation which are required to break down lipoprotein-containing surfactant. In addition, PPARγ downregulates M-CSF expression in alveolar macrophages in an NF-κB dependent manner [73]. Thus, the alveolar type II epithelial cells produce the niche signal which is necessary for alveolar macrophage development, which in turn contribute to alveolar homeostasis through clearance of surfactant produced by alveolar type II epithelial cells. The dominant role of the niche in imprinting has been shown in several other adoptive transfer studies. For instance, two studies showed that transfer of large peritoneal macrophages to the alveolar space causes these cells to gain CD11c expression [74] and partially shift their transcriptional program towards that of an alveolar macrophage [18]. However, it was not shown whether these cells are functionally equivalent to alveolar macrophages and if they can survive and proliferate. Other research has shown that this conversion is very inefficient and that this is possibly the result of the reduced plasticity of matured tissue-resident macrophages [71]. Note that PU.1 expression by alveolar macrophages is also regulated by the presence of GM-CSF and is proposed to play a critical role in the maintenance of lung homeostasis and innate immune host defense [75].

Although the essential role of the GM-CSF/PPARγ axis in alveolar macrophage development is well characterized, additional signals and TFs are involved in the development and functional maturation of alveolar macrophages. Recent research has shown that autocrine TGFβ signaling is critical for alveolar macrophage differentiation, maturation and maintenance through upregulation of alveolar macrophage signature genes, including PPARγ [76]. In addition, the alveolar macrophage population of BACH2-deficient mice display alterations in lipid handling, resulting in the development of alveolar proteinosis, whilst maintaining normal expression of genes involved in GM-CSF signaling [77]. Research by the same group has shown that the degree of pulmonary alveolar proteinosis is more severe in *Bach1/Bach2* double KO mice, but absent in *Bach1* KO mice [78]. In addition, the alveolar macrophages of *Bach1/Bach2* double KO mice have a more immature phenotype based on expression of F4/80, CD11b and Siglec-F. Finally, CEBPβ might also play a role in alveolar macrophage development and/or survival as the alveolar macrophage population in *Cebpb*-deficient mice is strongly reduced [58].

### 3.6 Osteoclasts

Osteoclasts are large multinucleated macrophages derived from granulocyte-macrophage progenitors that fulfill different functions, among them the resorption of bone matrix produced by osteoblasts, thereby maintaining bone homeostasis [79]. One of the major transcription factors involved in osteoclast development is NFATc1 [80], [81]. The crucial role of NFATc1 in osteoclast development was first shown in *Nfatc1*^−/−^*;Tie2-Nfatc1* mice [82]. These mice develop osteopetrosis, a disease characterized by an abnormally dense bone structure, caused by a deficiency in osteoclastogenesis. NFATc1 expression is regulated by RANKL which exists in two forms, a membrane bound form present on the surface of osteoblasts and a secreted form resulting from proteolytic cleavage of the membrane bound protein or alternative splicing [83], [84]. Osteoclastogenesis is a process that has been extensively studied. This process is based on the RANK/RANKL/OPG system that leads to the activation of NFATc1, the master regulator of osteoclast differentiation [85], [86]. Osteoclast differentiation starts with the recognition of the osteoblast produced RANKL by its receptor RANK found on the cell surface of osteoclast precursors. This binding leads to the recruitment of the TRAF6-TAB2-TAK1 complex which in turn leads to the activation of MAPK [87], NF-κB [88], [89] and JNK1 [90] signaling pathways in cooperation with M-CSF signaling [91]. The importance of M-CSF in osteoclast development was first shown in *Csf1^op/op^* mice, which suffer from congenital osteopetrosis due to a severe deficiency in osteoclasts [92]. Together with NFATc2, NF-κB activates NFATc1 expression which is then maintained through auto-amplification [93]. In resting state, NFATc1 is phosphorylated. In order to translocate into the nucleus, NFATc1 is subsequently dephosphorylated by calcineurin, a phosphatase activated by the calcium/calmodulin signaling pathway [94]. More specifically, the calmodulin CaMKIV has been shown to be critically involved in osteoclast differentiation and function [95]. In addition, NFATc1 expression is epigenetically regulated through histone acetylation and H3K27me3 demethylation in response to RANKL, thereby enhancing its auto-regulatory capacity [96]. Once in the nucleus, NFATc1 forms an osteoclast-specific complex containing AP-1 (c-Fos/c-Jun complex) [97], PU.1 [98], pCREB and MITF. This complex then drives expression of osteoclast signature genes involved in acidification and matrix degradation, such as tartrate-resistant acid phosphatase (*Trap*) [80], calcitonin receptor (*Ctr*), cathepsin K (*Ctsk*) [99], ATP6i [100] and β3 integrin (*Itgb3*) [101]. Concomitant to RANKL secretion, osteoblasts also produce osteoprotegerin (OPG), a soluble decoy receptor that binds RANKL and thus inhibits osteoclast differentiation [102]. Hence, osteoclastogenesis is a process which is tightly and specifically regulated by the osteoblast population.

Next to the RANK/RANKL/OPG system, osteoclast development also depends on TGFβ signaling [103]. The cytokine TGFβ is highly enriched in bone matrix and was shown to synergize with RANKL for induction of osteoclast-like cells *in vitro* [104]. Conversely, inhibition of TGFβ signaling almost completely abolishes RANKL-induced osteoclastogenesis [103]. Additionally, TGFβ1 suppresses apoptosis of osteoclast-like cells [104]. The interaction between both signaling pathways is mediated by SMAD3 which binds to TRAF6 upon recruitment to RANK [103].

Recently, a role for Retinoid X Receptors (RXRs) in osteoclast proliferation, differentiation and activation has been described [105]. Loss of RXR in osteoclast progenitors leads to the formation of abnormally large, multinucleated, non-resorbing osteoclasts [105]. In steady state, RXR homodimers controls *Mafb* expression in osteoclast progenitors. Loss of RXR results in an increased expression of *Mafb* and an impaired response to M-CSF, leading to less proliferation and abnormal organization of the cytoskeleton. In addition, NFATc1 is downregulated in RXR-KO osteoclast progenitors, suggesting that there might be an interaction between RXR and NFκB signaling in osteoclast differentiation.

### 3.7 Microglia

Microglia are the tissue-resident macrophages of the central nervous system (CNS). They are involved in maintaining CNS homeostasis by probing the brain parenchyma with their highly motile dendrites. In addition, they play a role in neuronal proliferation, development and synapse formation [106], [107], [108]. All microglia are derived from yolk sac macrophages [27]. This unique composition might be explained by the fact that the blood-brain barrier is formed, and thereby closes the CNS niche, before fetal liver monocytes are seeded in the tissues, thereby preventing their entry [109].

One of the major niche signals involved in microglial development is TGFβ, a cytokine expressed in the developing CNS of the embryo and by most cells in the CNS during adulthood [110]. Mice which lack TGFβ in the brain have a significantly reduced microglia population [111]. In addition, TGFβ is required to upregulate microglia signature genes *in vitro* [111]. Furthermore, the combination of M-CSF and TGFβ upregulates more microglial genes in *in vitro* cultured macrophages than M-CSF alone, suggesting that both signals are essential for microglial development [111]. However, it was recently shown that TGFβ signaling is not required for microglia survival, but rather to keep them in an inactivated state [112]. As a consequence of their activation, deletion of TGFβ signaling in microglia is accompanied by an altered surface phenotype with differential expression of key markers such as CD45, F4/80, MHCII and Siglec-H. This quiescent state is regulated by SMAD2/3 activation through TGFβ-dependent phosphorylation [113]. Next to TGFβ signaling, different groups proposed CSF1R signaling to be more important for microglia homeostasis in the adult brain as deletion of this signaling induces a rapid loss of microglia [27], [112], [114]. However, *Csf1^op/op^* mice, which have an inactivating mutation in the *Csf1* gene [92], exhibit a moderate reduction of the microglia population and only in distinct regions of the brain, indicating that an alternative mechanism exists to compensate for the absence of M-CSF [115], [116]. CSF1R can also bind IL-34 which was shown to induce macrophage survival, proliferation and differentiation *in vitro* [117]. In line with this, deletion of IL-34 considerably impairs microglia development in specific brain regions that do not overlap with those of the *Csf1^op/op^* model. This differential spatial loss of microglia can be explained by the distinct spatial expression pattern of M-CSF and IL-34, both expressed by neurons [118], that are expressed in distinct regions, thus indicating a non-redundant function of these two cytokines [24].

Recently, SALL1, a zinc finger transcription factor, has been identified as a microglia-specific transcription factor [112]. SALL1-deficient microglia have a reduced expression of microglia signature genes and upregulate genes associated with other macrophage populations, indicating that SALL1 is important in microglia development and the maintenance of microglia identity [112]. Interestingly, microglia signature genes, including *Sall1*, are lowly expressed in neonates. After birth, the expression of these genes gradually increases and reach maximum expression between 3 weeks and 2 months [111]. This might be explained by the fact that M-CSFR ligands are maximally expressed during postnatal brain development [118]. In addition, monocyte-derived microglia-like cells do not express SALL1, making SALL1 a specific marker for embryonically-derived microglia [112]. Moreover, this shows that both ontogeny and microenvironment influence the mature macrophage profile. Despite the importance of SALL1 in microglia development and function, the niche signal that drives its expression remains unknown. Intriguingly, recent research has shown that the gut microbiota is involved in microglia homeostasis through the production of short chain fatty acids. [119], [120]. As the gut microbiome changes after weaning (around 3–4 weeks after birth) [121], there might be a link with the increasing expression of SALL1 and other microglia signature genes after birth.

### 3.8 Langerhans cells and intestinal macrophages

Langerhans cells (LCs) are the bone marrow-derived mononuclear phagocytes of the epidermis which are involved in the uptake and transport of antigens to the skin-draining lymph nodes [122], [123], [124]. Due to this function, they are often categorized as DCs. However, unlike DCs, the LC population can self-maintain through proliferation in steady state conditions [125]. LCs require IL-34, produced by keratinocytes [24], and TGFβ1 for their development [126]. TGFβ1 is expressed by both keratinocytes and LCs, but only the autocrine TGFβ1 signaling is essential for LC development [127]. TGFβ induces the expression of RUNX3 and ID2, which are required for Langerhans cell maintenance [128], [129]. A more recent study has shown that inducible loss of TGFβ1 leads to a synchronized wave of LC migration to the lymph nodes [130]. Therefore, the absence of LCs in the epidermis of TGFβ1-deficient mice might be the result of a constant migration of newly formed LCs.

TGFβ signaling is also important for monocyte differentiation into colonic macrophages [131] and may also function through RUNX3, as this TF is highly expressed in intestinal macrophages [18]. In this same study, it was shown that TGFβ signaling regulates the expression of genes involved in Notch signaling, including *Hes1* and *Dtx4* [131].

### 3.9 Adipose tissue macrophages

At steady state, adipose tissue macrophages represent around 5% of total adipose tissue cells but increase up to 50% in obese mice, suggesting a potential role for this population in obesity [132]. This expansion has been linked to both local proliferation of resident macrophages and CCL2-mediated recruitment of monocytes followed by their differentiation into macrophages [133], [134]. However, the ontogeny of adipose tissue macrophages at steady state is still unclear. This macrophage subset fulfills different functions in the tissue such as removal of dead adipocytes, regulation of adipocyte lipolysis, storage of excessive adipocyte-released lipids as well as its gradual release into the bloodstream [135].

Recently, the heterogeneity of this population has been highlighted. Pirzgalska et al. have shown that some macrophages are found to be in close interaction with sympathetic neurons in the white adipose tissue [136]. This subset of neurons produces, among others, norepinephrine (NE), a neurotransmitter mediating lipolysis and fat mass reduction. Unlike other adipose tissue macrophages and consistent with their location, sympathetic neuron-associated macrophages express two proteins involved in the import and the degradation of NE allowing its regulation in the extracellular space: the NE transporter SLC6A2 and the NE catalyzer monoamine oxidase A. However, they have shown that this macrophage population plays a major role in obesity because specific ablation of SLC6A2 in these macrophages induced weight loss and lipid mobilization. Interestingly, they also highlight the presence of similar macrophages in the brown adipose tissue, where these macrophages are found in close interaction with nerve fibers and act as a NE sink [136].

In correlation with the latter finding, another group has confirmed that brown adipose tissue macrophages play a major role in obesity. Rett syndrome is a neurodevelopmental disorder caused by mutations in the gene encoding methyl-CpG binding protein 2 (MECP2). Wolf et al., have shown that MECP2 deficiency in brown adipose tissue macrophages results in spontaneous obesity due to a reduction of the brown adipose tissue sympathetic innervation and thus of the NE tissue levels which ultimately leads to an altered thermogenesis [137]. How this population of macrophages is regulating the tissue innervation remains to be elucidated, but these studies showed that the genetic profile of macrophages is finely shaped by their niche to perform functions that are essential for tissue homeostasis.

## 4 Refining the niche hypothesis: intra-organ local and inter-organ similar niches

Besides their immune functions, macrophages are actively contributing to the homeostasis of their tissue of residence by fulfilling functions that are strictly related to the latter. This implies that there is a strong imprinting by the tissue, which shapes the macrophage genetic program into a specific macrophage population. Of note, the niche signals should not be restricted to the presence in one or other organ. Indeed, different macrophage populations can be found in the same organ, suggesting the existence of distinct intra-organ niches which provide different niche signals.

For a long time, the liver was considered to harbor only one tissue-resident macrophage population, i.e. Kupffer cells. However, a new population of liver-resident macrophages, called liver capsular macrophage, has recently been described by Sierro et al. [138]. Although seeding the same organ, these two populations are distinct in term of location and function. Indeed, while Kupffer cells are located within the liver sinusoids and participate in liver metabolic functions, the newly described subset of macrophage is located along the hepatic capsule and is involved in the immune response against peritoneal pathogens that access the liver’s outer membrane [64], [65], [138]. Of note, unlike Kupffer cells this macrophage population is derived from Ly6C^hi^ monocytes and start to accumulate in the liver around weaning. Thus, one could argue that their origin shapes their location and functions. However, it has been shown that specific depletion of Kupffer cells leads to the replenishment of the population by Ly6C^hi^ monocytes that will rapidly differentiate into Kupffer cells that are transcriptionally and functionally identical to embryonically-derived Kupffer cells [64].

The gut is another striking example. To avoid development of auto-immune disease and food allergies, the immune system has to be tolerant to commensal bacteria and food antigens. However, the gut is also an important entry site for a broad spectrum of pathogens. To solve this problem, the gut is divided into sites specialized either in nutrient absorption (villi) or in pathogen detection (Peyer’s patches), each of them colonized by different macrophage populations that are fully adapted to their micro-environment. Indeed, villi macrophages are strongly involved in the establishment of a tolerogenic environment through the release of anti-inflammatory cytokines, such as IL-10 [139]. Unlike the former, Peyer’s patch macrophages are unable to release IL-10 after stimulation but instead will produce pro-inflammatory cytokines such as IL-6 and TNF [140], [141]. Furthermore, the IL-10 signaling pathway is downregulated in Peyer’s patch macrophages compared to villi macrophages [142], [143]. Contrary to the liver, this could not be attributed to their origin as both populations are continuously replaced in adult mice by Ly6C^hi^ monocytes, but rather to their micro-environment as the surface of villi is protected by physical and chemical barriers that are almost absent on Peyer’s patch surface [8], [140], [141], [144], [145]. The epithelium of Peyer’s patches is also enriched in a particular cell type specialized in the transport of microorganism from the gut lumen to the Peyer’s patch, contributing to the stimulation of Peyer’s patch macrophages [146].

In the brain, microglia are the most abundant macrophage population and are found to be in close interaction with neurons in the parenchyma. Through secreted and membrane bound signals such as CD200 and CX3CL1, neurons are able to keep microglia under a quiescent state while a reduction in these factors can induce their reactivation [147], [148]. Thus, by their constant communication with microglia, neurons are able to shape the phenotype of this macrophage population. Three other brain macrophage population have been described and are located in different CNS niches. In detail, (1) perivascular macrophages are located between endothelial and glial basement membranes, (2) subdural meningeal macrophages are closely associated to ER-TR7+ fibroblast-like cells lining the meninges and meningeal vasculature and finally (3) choroid plexus macrophages are located in the stroma and epithelial layer of the choroid plexus [149], [150]. Through RNA-seq analysis, Goldmann et al., have shown that although microglia and perivascular macrophages are transcriptionally closely related as compared to monocytes and peritoneal macrophages, which might be due to an organ-specific effect, they retain some cell-specific differences [151]. Furthermore, while all brain macrophage populations are embryonically-derived, only choroid plexus macrophages are partially renewed by Ly6C^hi^ monocytes during adulthood and exhibit a shorter turnover. These differences among brain macrophage subsets might be the result of local CNS environmental cues.

Opposite to intra-organ local niches, the notion of similar niches in different organs may add an extra layer of complexity to the concept. One could argue that a similar niche provides similar niche signals to developing macrophages. For example, some macrophages can be found in close interaction with nerve cells, such as sympathetic neuron-associated macrophages in the adipose tissue, nerve-associated macrophages in the cornea, microglia with neurons in the brain or even muscularis macrophages with enteric neurons in the gut [136], [152], [153], [154]. Thus, it cannot be excluded that muscularis macrophages and microglia for example, may share part of their genetic profile because of their proximity to nerve cells. In line with this, Gautier et al. have shown by a hierarchical clustering analysis based on macrophage-core signature a close transcriptional relationship between gut macrophages and microglia as compared to other tissue-resident macrophages [37]. Future research will have to point out to what extent these different macrophages are functionally related and if their respective niches contain similar signals that induce the same signaling pathways in these macrophages.

## 5 The importance of nature and nurture in functional specialization

As mentioned above, macrophage can be embryonically-derived and monocyte-derived and all precursors have the potential to differentiate into the same type of tissue-resident macrophage when given the opportunity as was shown in the lung [71]. In other words, nurture rather than nature seems to play a dominant role in macrophage development. The question remains whether these macrophages are completely identical. For instance, a recent study has shown that monocytes which infiltrate the alveolar space after gammaherpesvirus infection can differentiate into long-lived alveolar macrophages that inhibit house dust mite-induced allergy [155]. In this case, nature seems to play a significant role in the function of alveolar macrophages. However, it is possible that the niche is (permanently) affected due to the viral infection, causing the monocytes to receive a different imprinting during their development into alveolar macrophages. Additional evidence for the importance of nature is found in the CNS. Buttgereit et al. have shown that SALL1, a microglia-specific transcription factor, is not expressed by monocyte-derived microglia after microglia depletion [112]. This indicates that expression of SALL1 is inherent to embryonically-derived microglia and is not induced by niche signals. As discussed in a recent opinion paper by Bonnardel & Guilliams, the interplay between nature and nurture in steady state and during inflammation will require novel “functional” fate-mapping tools [156].

## 6 Conclusion and future perspectives

In this review, we gave an overview of the major lineage-determining TFs that establish the core macrophage program and the niche signals and signal-dependent TFs which drive tissue-resident macrophage development by adapting the core macrophage program to meet tissue-specific needs. These signal-dependent TFs are often referred to as being essential for tissue-resident macrophage development. However, it is important to note that functional specialization during macrophage development is often essential for their survival in their respective niche. By contrast, it is possible that a signal-dependent TF is important to acquire a specific function which is part of its identity, but does not affect its survival in steady state conditions.

Despite the heterogeneity of tissue-resident macrophage populations in the body, certain common features in their development can be appreciated. For instance, macrophage development is driven by an interplay between multiple niche signals, signaling pathways and TFs. Conversely, certain signaling pathways are involved in the development of multiple tissue-resident macrophage populations. Intriguingly, there is often a functional relationship between the cell that produces the niche signal and its corresponding tissue-resident macrophage. This is most apparent in the lung, bone and brain. Recently, such a functional module consisting of a fibroblast and a macrophage was generated *in vitro* [157].

Until now, only one or a small number of TFs and associated niche signals have been described for some tissue-resident macrophages. These were often discovered by studying KO models with a severe reduction of a particular tissue-resident macrophage population. Taking into account the close relationship between development, functional specialization and survival, it is possible that certain functional specializations are only important when homeostasis is disturbed. Conversely, a seemingly unaffected population does not mean the tissue-resident macrophages have reached functional maturity, since we often use only a limited number of general macrophage markers to identify tissue-resident macrophage populations. Therefore, TF KO models should be studied in steady state and under a number of relevant disease conditions using an integrated approach. This may include the measurement of relevant physiological parameters, (single cell) RNA-seq, multi-parameter flow cytometry and mass cytometry. Consequently, generation of such multidimensional datasets will increase the need for bio-informatics tools [158], [159].

These tools will also help us identify unique tissue-resident macrophage markers, such as CLEC4F in Kupffer cells [64], which will allow us to better understand the mechanisms involved in tissue-resident macrophage development. Until now, tissue-resident macrophage populations are still identified based on common markers such as F4/80 and CD64, which poorly define macrophage diversity in the body. Using these macrophage-specific markers, new macrophage-specific Cre lines can be created to knock-out signal-dependent TFs of interest to study their effect on the macrophage function in homeostasis and disease conditions. In addition, niche cell-specific Cre lines will allow us to knock-out genes involved in niche signaling to the developing tissue-resident macrophage. Using both systems, a direct and specific connection between niche cell, niche signal and developing tissue-resident macrophage can be made.

Once we have elucidated the regulation of tissue-resident macrophage development under steady state conditions, it will be interesting to investigate how inflammation or cancer affects the niche signals that instruct the development of monocyte-derived macrophages which play an important and often dual role in these diseases. Consequently, this may pave the way for the development of therapies involving compounds that block or induce certain signal-dependent TFs, thereby allowing us to modulate the function of macrophages.
